# Ameliorative Effect of Citrus Lemon Peel Extract and Resveratrol on Premature Ovarian Failure Rat Model: Role of iNOS/Caspase-3 Pathway

**DOI:** 10.3390/molecules28010122

**Published:** 2022-12-23

**Authors:** Maysa A. Mobasher, Marwa T. Hassen, Rasha A. Ebiya, Norah A. Alturki, Ahmad Alzamami, Hanaa K. Mohamed, Nabil S. Awad, Dina Khodeer, Bosy A. Abd El-Motelp

**Affiliations:** 1 Department of Pathology, Biochemistry Division, College of Medicine, Jouf University, Sakaka 72388, Saudi Arabia; 2 Department of Zoology, Faculty of Women for Arts, Science and Education, Ain Shams University, Cairo 11757, Egypt; 3 Clinical Laboratory Science Department, College of Applied Medical Science, King Saud University, Riyadh 11433, Saudi Arabia; 4 Clinical Laboratory Science Department, College of Applied Medical Science, Shaqra University, Al Quwaiiyah 11961, Saudi Arabia; 5 Department of Genetics, Faculty of Agriculture and Natural Resources, Aswan University, Aswan 81528, Egypt; 6 College of Biotechnology, Misr University for Science and Technology, Giza 12563, Egypt; 7 Department of Pharmacology & Toxicology, Faculty of Pharmacy, Suez Canal University, Ismailia 41522, Egypt

**Keywords:** cyclophosphamide, lemon peel extract, resveratrol, premature ovarian failure, inflammatory marker, gene expression

## Abstract

Premature ovarian failure (POF) is described as a loss of oocytes and the absence of folliculogenesis and is considered an adverse effect of chemotherapeutic drugs, which leads to infertility. Subsequently, the existing inquiry was achieved by exploring the potential suspicious influences of lemon peel extract (LPE), and resveratrol (RES) on cyclophosphamide (CPA) induced-POF. The results showed that CPA-induced POF significantly decreased serum estradiol (E2) and progesterone levels, along with a considerable rise in serum luteinizing hormone (LH) and follicle-stimulating hormone (FSH) levels. Moreover, CPA administration to rats significantly increased the serum level of Malondialdehyde (MDA) and significantly lowered the levels of reduced glutathione (GSH) and superoxide dismutase (SOD); in addition, it increased nuclear factor kappa B (NF-κB) levels, tumor necrosis factor-α (TNF-α), as well as cyclooxygenase 2 (COX-2) with the spread expression of inducible nitric oxide synthase (iNOS) mRNA levels and caspase-3 (Casp3) levels in ovarian tissues versus the control rats. However, treatment with LPE and RES suppressed the triggering of NF- κB pathways, evidenced by a considerable reduction in Casp3 & iNOS mRNA expression level and significant ameliorative effects in all evaluated parameters, as confirmed by the histological and immunohistochemical investigation when comparing the model group. In overall findings, both lemon peel extract and resveratrol can mitigate the adverse effects of CPA-induced POF. Most crucially, its combination therapy is a promising pharmacological agent for this disease.

## 1. Introduction

Premature ovarian failure (POF) is described as ovarian dysfunction in women under the age of 40 and is distinguished by ovarian tissue degeneration, long-term amenorrhea, and infertility [1]. Also, POF is characterized by menstrual disruption, decreased estrogen levels, increased gonadotropin levels, and high follicle-stimulating hormone (FSH) [2]. POF’s possible causes could include genetic defects., immunological diseases, chemotherapy, and radiotherapy [3]. Cyclophosphamide (CPA), an alkylating and immunosuppressive agent, is one of the chemotherapeutic drugs that have irreversible cytotoxicity on major internal organs, especially the ovary. CPA induces ovarian failure, which produces severely damaging alterations in the brain and medulla [4]. CPA may promote apoptosis in granulosa cells by destroying their DNA, leading to follicular degeneration, which reduces estradiol and progesterone release while increasing gonadotropin secretion from the anterior pituitary, ultimately resulting in poor ovarian reserve (POR) [5]. Its cytotoxicity is also caused by the production of reactive oxygen species (ROS), leading to lipid peroxidation, mitochondrial dysfunctions, caspase activation and apoptosis, and inflammation caused by various inflammatory mediators [6].

*Citrus limon* (Lemon) is a medicinal plant that is appropriate to the Rutaceae family. Lemons are abundant in citric acid, minerals, ascorbic acid, flavonoids, and essential oils [7,8]. The fruit contains a high concentration of phenolic chemicals, including flavonoids such as hesperidin, naringin, and naringenin and flavones such as diosmin [9]. These compounds exhibit many pharmacological actions in the body and are scientifically confirmed to confer positive health effects to limit the developing of various illness due to the abundance of vitamins, minerals, and other active ingredients related to antioxidant, anti-inflammatory, antiatherosclerotic, antiseptic, antifungal, anticarcinogenic, antimutagenic and hepatoprotective actions, and they boost the body’s immune and cardiovascular system [10,11]. Citrus fruits can protect the human body from injury caused by ROS by reducing the loading of free radicals and/or enhancing the endogenous antioxidant protection system [12].

It has been observed that resveratrol (RES) reduces inflammation and possesses, antioxidant, antiproliferative, proapoptotic, anticancer, neuroprotective, antiviral, and anti-aging actions [13]. It has direct ROS scavenging activities and can enhance endogenous antioxidant cellular mechanisms. It is a transcriptional factor that is mediated by the phytoestrogen-modifying estrogen receptor (ER) [14]. Several studies reported that RES enriches ovarian function in a rat model of POF through its ability to reduce inflammation, which are mediated by various molecular targets, such as the silencing information regulator 1 (SIRT1) [15,16]. By promoting the production of SIRT1 in ovarian granulosa cells, SIRT1 activation limits NF-κB activity and, consequently, decreases proinflammatory cytokines [17]. Resveratrol increases progesterone production and luteinization-related gene expression in theca cells [18].

Therefore, the goal of this investigation was to inspect the effects and underlying mechanisms of Citrus Lemon Peel Extract (LPE) and RES on experimental rats with cyclophosphamide-induced Premature Ovarian Failure, as well as their current therapeutic and preventive strategies focusing on the POF.

## 2. Results

### 2.1. Effect of LPE and RES on the Serum Hormonal Markers of Cyclophosphamide-Treated Rats

**Figure 1:**
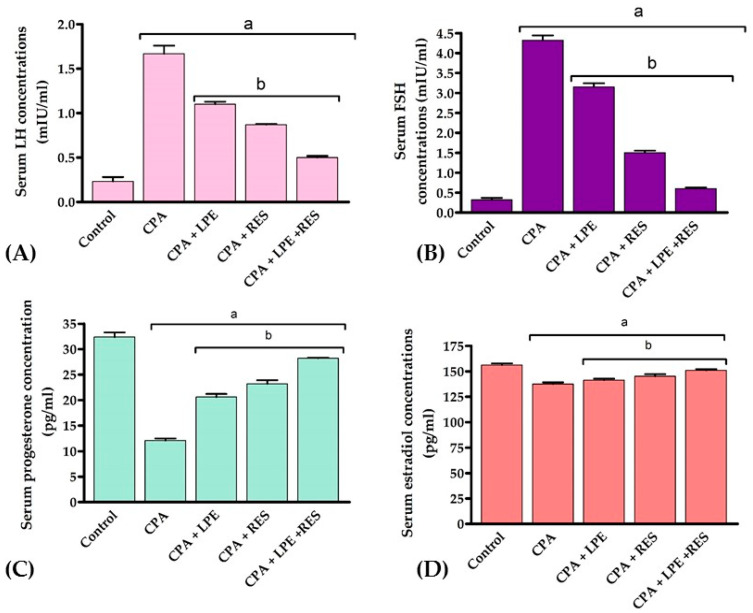
Effects of LPE and/or RES on the serum hormonal markers of cyclophosphamide-treated rats. (**A**) LH concentration, (**B**) FSH concentration, (**C**) Progesterone concentration, (**D**) Estradiol concentration. Data expressed as mean ± SEM, a *p* < 0.05 versus the control group; b *p* < 0.05 versus a CPA group, (n = 7). CPA: Cyclophosphamide, LPE: Lemon peel extract, RES: resveratrol.

The findings in Figure 1 show that CPA significantly augmented the levels of FSH and LH (*p* < 0.05) with significantly decreased estradiol and progesterone levels (*p* < 0.05) when compared with the control group. However, the treatment of CPA rats with LPE and RES produced a considerable improvement (*p* < 0.05) in FSH, LH, estradiol, and progesterone levels compared to the CPA group. Moreover, the combination therapy of LPE plus RES led to more favorable effects on all these hormones when compared with the CPA group. These findings revealed that co-treatment of both LPE with RES in CPA-treated rats alleviated the hormonal disturbance caused by CPA.

### 2.2. Effect of LPE and RES on the Serum Antioxidants and Oxidative Stress Markers of Cyclophosphamide-Treated Rats

**Figure 2:**
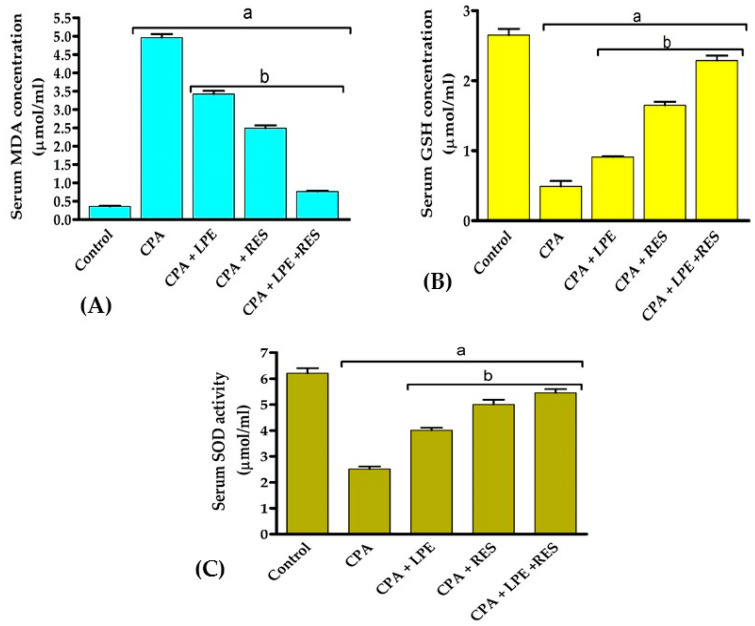
Effects of LPE and/or RES on the antioxidants and oxidative stress markers in cyclophosphamide-treated rats’ ovarian tissue. (**A**) The levels of MDA, (**B**) GSH, and (**C**) SOD enzyme activity. Data expressed as mean ± SEM. a *p* < 0.05 versus control group; b *p* < 0.05 versus a CPA group, (n = 7). CPA: Cyclophosphamide, LPE: Lemon peel extract, RES: resveratrol.

Figure 2 reveals that the levels of MDA were significantly upregulated (*p* < 0.05) with a large drop in GSH level and SOD activity after CPA treatment contrasted with a negative group. Treatments with LPE, RES, or LPE + RES significantly downregulated (*p* < 0.05) the MDA levels while significantly elevating the GSH level and SOD activity, compared with the CPA group. These results showed that co-treatment of both LPE with RES in CPA-treated rats ameliorated the oxidative stress caused by CPA treatment.

### 2.3. Effects of LPE and RES on Ovarian Tissue Inflammation Markers in Cyclophosphamide-Treated Rats

**Figure 3:**
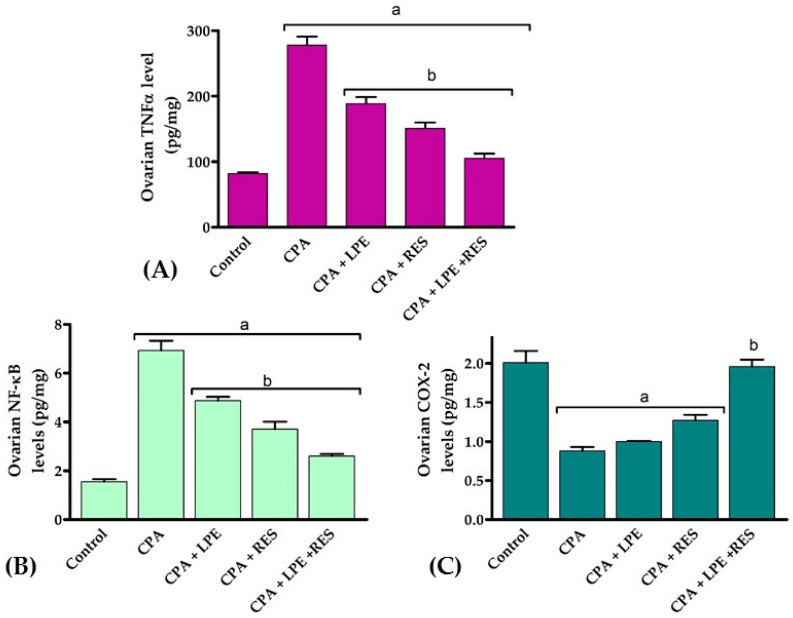
Effects of LPE and/or RES on ovarian tissue inflammation markers in cyclophosphamide-treated rats. (**A**) TNF-α levels, (**B**) NF-κB and (**C**) COX-2 levels. Data expressed as mean ± SEM. a *p* < 0.05 versus control group; b *p* < 0.05 versus a CPA group, (n = 7). CPA: Cyclophosphamide, LPE: Lemon peel extract, RES: resveratrol.

TNF-α, NF-KB, and COX-2 levels in CPA rats have been substantially (*p* < 0.05) higher than in normal control rats, as shown in Figure 3. After treating with LPE and RES, the values were considerably (*p* < 0.05) lower in all further groups. Furthermore, the ovarian levels of TNF-α, NF-KB, and COX-2 were also effectively decreased (*p* < 0.05) in rats cured by LPE + RES compared to untreated CPA rats. From these data, we discovered that co-treatment of both LPE with RES in CPA-treated rats minimized the inflammation caused by CPA treatment.

### 2.4. Histopathological Investigations

**Figure 4:**
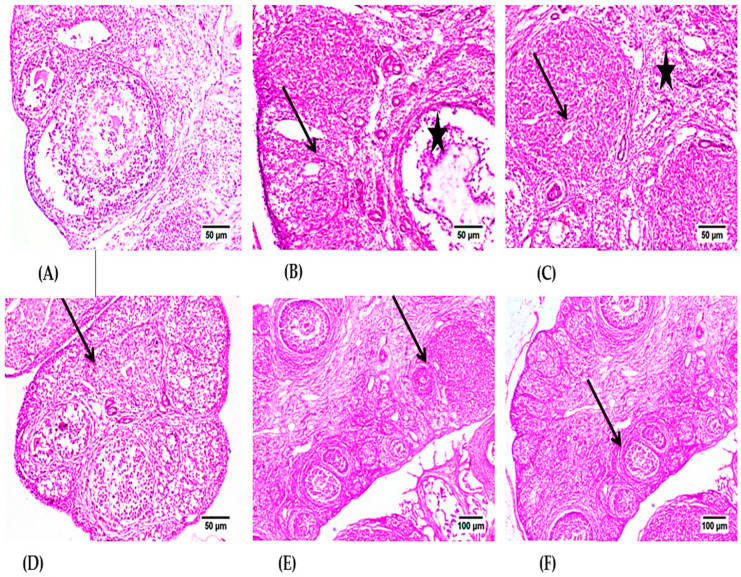
Photomicrographs of H- and E-stained ovarian tissue sections of the control and experimental groups. (**A**) control group, showing normal Graafian and secondary follicles. (**B**) CPA-treated group showing luteal structure (arrow) and Graafian follicle (star) with mild mononuclear inflammatory cell infiltration. (**C**) CPA-treated group showing the luteal structures (stars) and the intense interstitial inflammatory reaction (arrow). (**D**) The LPE-treated group displays various stages of progressing follicles with the existence of inflammatory cell infiltration. (**E**) The RES-treated group showed normal developing follicles with mild inflammatory edema. (**F**) The RES + LPE-treated group showed apparently normal ovarian tissue. CPA: Cyclophosphamide, LPE: Lemon peel extract, RES: resveratrol.

Microscopic examination of ovaries from the control group (Figure 4A) revealed normal histology of the ovary that showed numerous corpora lutea with multiple growing follicles in different stages. Examination of the CPA group (Figure 4B,C) revealed interstitial cell hyperplasia of variably sized clusters of pale-staining cells that were interspersed among several follicles in various stages of development, including large atretic follicles, mature corpora lutea, and congested blood vessels. Large polyhedral cells with pale eosinophilic granular cytoplasm and central nuclei characterized the hyperplastic interstitial cells. The corpus luteum showed degenerated and necrotic cells in the CPA group in some instances. The section of ovaries in the CPA with either LPE or RES groups (Figure 4D,E) showed a slight improvement in the corpus luteum. Furthermore, the CYP group treated with the LPE + RES (Figure 4F) displayed normal follicles in different stages. Our histological examination revealed that co-treatment of both LPE with RES in CPA-treated rats restored the architecture of ovarian tissues nearly to the control group.

### 2.5. Immunohistochemical Analysis 

**Figure 5:**
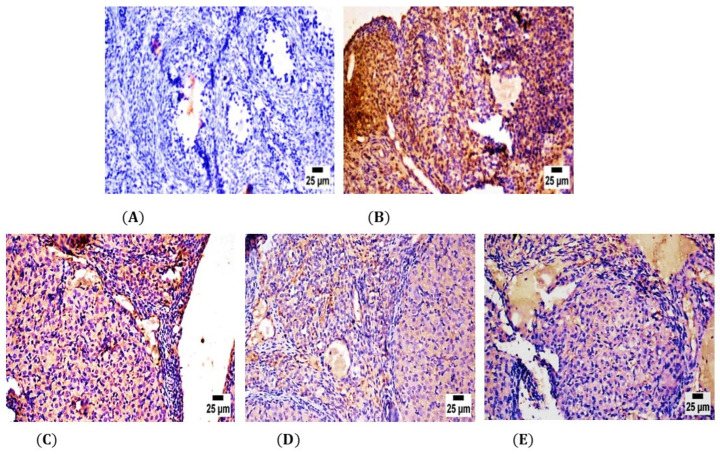
Photomicrographs of the ovarian tissue sections of the control and experimental groups. (**A**) showing negative secretion of TNF-α polyclonal antibodies in the self-control group. (**B**) The CPA-treated group showed strong positive expression of TNF-α polyclonal antibodies. (**C**) The LPE-treated group showed moderate expression of TNF-α polyclonal antibodies. (**D**) RES group showed lower expression of TNF-α polyclonal antibodies. (**E**) LPE + RES showed minimal production of TNF-α polyclonal antibodies. CPA: Cyclophosphamide, LPE: Lemon peel extract, RES: resveratrol.

In the control group, the ovary tissue showed negative reactivity (0) for TNF-α polyclonal antibodies in Graafian follicles and luteum (Figure 5A); however, in the CPA group, the ovaries showed high expression of TNF-α polyclonal antibodies in Graafian follicles and luteum contrasted to the self-control group at *p* ≤ 0.05 (Figure 5B). Specifically, both the ovaries of LPE & RES groups showed moderate to mild cytoplasmic reactivity (++) for TNF-α polyclonal antibodies in Graafian follicles and luteum with a significant decrease in optical density % of TNF-α polyclonal antibodies in comparison with the CPA group at *p* ≤ 0.05 (Figure 5C,D). On the other hand, ovaries in the LPE + RES group showed improvement in weak cytoplasmic reactivity (+) for TNF-α polyclonal antibodies in Graafian follicles and luteum (Figure 5E) with significant inhibition in optical density % of TNF-α polyclonal antibodies compared with the CPA group. This immunohistochemical analysis affirmed the anti-inflammatory effects of co-treatment of both LPE and RES in CPA-treated rats.

### 2.6. Gene Expression Analysis

The mRNA-transcription levels of two different apoptotic and inflammatory molecular biomarkers; Casp3 and iNOS genes, respectively. These genes were quantified in ovarian tissues using real-time PCR utilizing GAPDH as a housekeeping gene. 

#### 2.6.1. iNOS Gene Expression Analysis 

**Figure 6:**
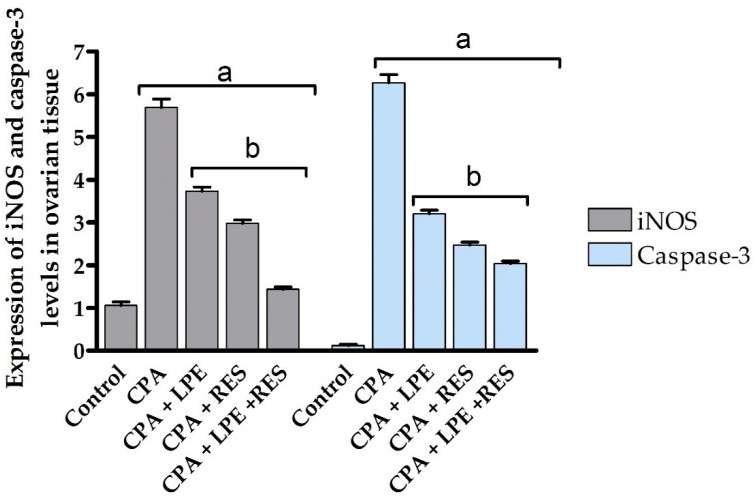
Effects of LPE and/or RES on the expression of iNOS and Casp3 levels in ovarian tissue of cyclophosphamide-treated rats. Data expressed as mean ± SEM. a *p* < 0.05 versus control group; b *p* < 0.05 versus a CPA group, (n = 7). CPA: Cyclophosphamide, LPE: Lemon peel extract, RES: resveratrol.

The inflammatory effect of POF induced by CPA administration established a significant upregulation (*p* ≤ 0.05) of the iNOS gene expression level compared to the control group. The protective role of LEP, as well as of RES separately, was detected by a significant (*p* ≤ 0.05) decline in iNOS gene expression level versus the CPA group, whereas co-treatment with LPE and RES showed significant downregulation in the levels of iNOS gene expression contrasted to the CPA-treated rats. Combining RES and LPE revealed the maximum anti-inflammatory ameliorative effect in the modulation of ovarian failure as compared to all tested groups (Figure 6). 

#### 2.6.2. Casp3 Gene Expression Analysis 

Casp3, an apoptotic molecular biomarker, showed maximum upregulation of mRNA expression after CPA administration compared to all studied groups. Treatment with LPE and RES after CPA administration resulted in a significant (*p* ≤ 0.05) decrease in casp-3 mRNA expression level compared to the CPA group (Figure 6). However, co-treatment with LEP and RES following CPA administration was more effective than treatment with LEP or RES separately, producing significant inhibition (*p* ≤ 0.05) of Casp3 gene expression at the mRNA level between all groups. All tested groups had statistically significant variance (*p* ≤ 0.05) between control, induction, and treated groups (Figure 6). These results indicated the anti-apoptotic effects of co-treatment of both LPE and RES in CPA-treated rats.

## 3. Discussion

The present study provides recent findings and highlights the possible molecular mechanism of lemon peel extract and resveratrol in ameliorating CPA-triggered premature ovarian failure in female rats. The present study discovered that CPA administration causes POF by worsening follicular atresia and impaired hormonal functions, which are associated with several mechanisms, including oxidative stress with lipid peroxidation, inflammation, and apoptosis of the ovarian follicles. These results were in agreement with those who noticed that CPA significantly increased LH and FSH together with deceasing estradiol and progesterone levels in POF rats [19,20]. The presence of these hormonal alterations is utilized as a sign of ovarian failure [21]. Moreover, some studies reported that oxidative stress damage in granulosa cells promotes apoptosis, which leads to ovarian follicle atresia after CPA treatment [22]. Otherwise, by encouraging oocyte apoptosis, CPA can indirectly accelerate the activation of primordial follicles. It can also increase primordial follicle growth via the phosphatidylinositol-3-kinase/protein kinase B(PI3K/PTEN/Akt) pathway, resulting in an accelerated lessening of primordial follicles and the initiation of POF [23,24].

Furthermore, FSH increases ovarian follicle growth and maturation by acting directly on granulosa cell-specific receptors. The combination of FSH and E2 levels is crucial for predicting POR [25]. Moreover, FSH can also help ovarian granulosa cells convert PI3K-Akt from just an inactive status to an activated conformation (p-Akt) [26]. The activation of phosphorylated Akt (p-Akt) in the granulosa cells of the ovaries boosted protein synthesis and maturity, allowing them to produce more estradiol. In accordance with the aforementioned results, our findings showed that the decline in estradiol and progesterone levels following CPA administration could be due to granulosa cell damage, which produces a decrease in FSH receptor expression in cells, limiting the influences of FSH. Simultaneously, the number of synthetic E2 sites decreases in addition to alkylating drugs’ blockage of progesterone receptors. These results were confirmed by [27] and Chinwe et al. [28], who reported that the decline in estradiol levels might result from estrogen regulation via suppression of its receptors and expression of the hormone.

Our findings demonstrated that treatment with either LPE, RES, or both enhanced ovarian reserve biomarkers following CPA-induced ovarian insufficiency. In contrast to those rats that received CPA, the LPE- and RES-treated groups showed large declines in FSH and LH levels, accompanied by considerable increases in estradiol and progesterone levels. Consistent with our results, Amin and Hamza [29] reported that plant alkaloids and flavonoids had been proven to lower LH and FSH levels in the blood. Therefore, the existence of these phytochemicals (flavonoids) in the LPE might give reasons for the improvements observed in our study. Furthermore, flavonoids from plant origin such as Naringenin, Hesperidin, and Rutin are reported to be helpful in the treatment of various diseases experimentally [30].

Furthermore, our findings demonstrated a significant decline in the FSH and LH levels connected with a large increase in estradiol and progesterone levels in the group treated with RES in contrast to those given CPA. Our findings could have been related to the beneficial impacts of RES on female rats’ reproductive functions [31]. Our findings are compatible with those of [32], who reported that RES protects female mice from POF by enhancing germline stem cells’ persistence. In addition, RES supplementation significantly improved uterine artery circulation and embryonic body mass in mice [33]. Moreover, RES is expected to affect the progress of a new therapy for polycystic ovary syndrome [34]. The present findings are consistent with a prior study that found RES augmentation gives protection and recovers the reserve of ovarian stroma and maturing ovarian follicles by the elevated FSH, LH, testosterone, estradiol, and progesterone levels in the immature uteri of rats with both control and chemotherapy groups [35,36]. 

Furthermore, our findings implied that giving CPA to normal rats produced a large rise in MDA levels and a significant decline in GSH and SOD levels comparable to those of normal rats. This could be due to CPA toxicity in producing different pathological abnormalities, including oxidative stress damage, apoptosis, and inflammation [37], thus, oxidative stress triggered by the deactivation and depression of antioxidant enzymes [38]. This result is consistent with those of [39], who attributed the elevation of MDA levels and the depletion in GSH and SOD levels to the interfering of acrolein with antioxidant defense systems, impeding nuclear and cytoplasmic development of oocytes and causing apoptosis are two ways in which oxidative stress can contribute to ovarian failure via impairment of CYP450.

There is an important mechanistic relationship between oxidative stress & POF [40]. Oxidative stress damages the intraovarian environment by slowing the nuclear and cytoplasmic maturation of oocytes and encouraging apoptosis, resulting in an imbalance between the generation and elimination of ROS. Therefore, the increase of ROS in the ovaries degrades oocyte quality, causes granulosa cell (GC) death, and results in rapid corpus luteum degradation [41,42,43]. In addition, the amplification of lipid peroxidation cascades causes oxidative damage to the ovary, which significantly impacts folliculogenesis, meiosis, and ovulation, finally leading to ovarian failure [44].

Treating CPA rats with LPE and RES, or in cooperation, contributed to large reductions in ovarian MDA levels and a large increase in ovarian levels of GSH and SOD in comparison to the CPA group. These findings align with those of [45,46]and found that LPE treatment was followed by a notable normalization of these parameters in rats. Furthermore, LPE showed improve in growth, antioxidant status, and immunological response, reducing oxidative stress and preventing free radical formation [47,48]. It has been discovered that phytocompounds, particularly flavonoids, have the ability to protect membranes and biological macromolecules from free radical damage [49]. In agreement with these results, the present findings showed that the flavonoid compounds present in LPE such as narirutin, hesperidin, and nobiletin, have the ability to reduce the oxidative stress induced by CPA in POF rats via enhancement of antioxidant status and reduction of lipid peroxidation levels. have been several previous studies of similar findings [10,50]. 

Conversely, RES has been shown in several studies to help protect ovarian tissue from oxidative damage due to its natural antioxidant characteristics [15]. It possesses antioxidant capabilities that can help improve endogenic cellular antioxidant defenses and direct ROS scavenging capabilities [51]. It has been claimed that the antioxidant activity of the selected plant extract is largely attributable to phenolic and flavonoid groups rather than to any single component because of the correlation of total phenolic and flavonoid content with an antioxidant capacity [52]. Furthermore, Özcan et al. [34] reported that antioxidants prevent follicular atresia caused by apoptosis and ROS-induced impairment. It has been demonstrated that RES increases the activity of natural cellular antioxidant systems such as SOD and catalase while inhibiting the production and activity of nicotinamide adenine dinucleotide phosphate (NADPH) oxidase sited in mitochondria [53]. Along the same line, Kong et al. [54] discovered that RES enhanced the overall quantity of oocytes, decreased atretic follicles, and prevented the transition from primordial to developed follicles and apoptosis. It has been reported that RES may stimulate ovarian activity and slow ovarian aging. In POF animal models, RES activates primordial follicles, increases the quantity of primary, primordial, and developing follicles, enhances granulosa cell proliferation, reduces ovarian inflammatory response, preserves the ovarian structure, and prevents oocyte and granulosa cell damage [55,56].

Moreover, the present study demonstrated a substantial elevation in the proinflammatory cytokine levels of TNF-α, NF-kB, and COX-2 with a consequent rise in the transcription gene of iNOS and Casp3 within the CPA group comparable to the control group. These findings could be explained by CPA’s enhanced storage in ovarian tissue and its potential to generate chronic inflammation by overexpressing the proinflammatory nuclear transcription factor NF-kB [57]. Correlating with our results, Ibrahim et al. [35] indicated that the accumulating of CPA active metabolites is thought to induce damage to several organs from oxidative stress by increasing the formation of ROS that degrade proteins and DNA in cells, thus causing inflammation and apoptosis [58,59]. Furthermore, iNOS results from activated macrophage transcriptional activity and is responsible for NO’s long-term and extensive synthesis [60]. Additionally, COX-2 is a transcriptional regulator that abundantly promotes inflammatory disorders, angiogenesis, and cancer cell invasion [61]. Overproduction of NO and prostaglandin E2 (PGE2) is caused by enhanced expression of iNOS and COX-2 [62]. Both the iNOS and COX-2 genes have NF-kB and AP-1 binding locations in their promoters. The transcription of target genes in the nucleus, including inflammatory genes such as COX-2 and iNOS, is activated by cytoplasmic transcription factors such as AP-1 and NF-kB, implying that these transcription pathways are implicated in the regulation of iNOS and COX-2 and the consequent formation of NO or PGE2 [62,63]. Likewise, CPA suppressed mitogen-activated protein kinase (MAPK), a potent anti-inflammatory molecule, resulting in large increases in IL-6, IL-1B, IL-2, and TNF-α [64,65]. CPA also promotes cell death by augmenting the amounts of proapoptotic enzymes, including Casp-3, while decreasing cell survival pathways [66]. NF-kB is an essential regulator of numerous genes responsible for inflammation, lymphocyte stimulation, cell proliferation, and apoptosis, and its activation is essential for downstream inflammatory mediators such as TNF-α and IL-6. NF-kB also contributes significantly to acute inflammatory processes and diseases associated with oxidative stress [67].

The current study showed the level of COX-2 in ovarian tissue increased significantly following treatment with CPA. These results are consistent with a similar study by Vyas et al. [68,69], who reported that inflammatory alterations play a crucial role in cisplatin toxicity, specifically the NF-κB pathway, which is triggered by cisplatin and then activates the transcription machinery of numerous inflammatory cytokines, such as TNF-α, and inflammatory enzymes, such as COX-2. Moreover, COX-2 is one of the NF-κB target genes, and it is controlled by growth factors and different cytokines such as IL1β, IL6, or TNF-α [61]. TNF-α has a well-known role in inflammatory disorders by activating COX-2, which can be exacerbated by oxidative stress [70]. Ricciotti and FitzGerald [71] mentioned that COX-2 accumulation is the reason for numerous processes of tissue injury through the synthesis of vasoactive and proinflammatory composites. 

Our finding demonstrated that the treatment with LPE, RES, or a combination of both resulted in a deterioration in the expressions of the inflammatory mediators TNF-α, NF-kB, and COX-2 with a downregulation in the expression of iNOS and Casp3levels in ovarian tissue after treatment with CPA. This result coincides with the findings of [72], who described citrus lemon fruit’s anti-inflammatory and protective effects, as citrus species contain three types of flavonoids: flavanones, flavones, and flavonols. Its anti-inflammatory actions are attributable to its prevention of the formation and activity of proinflammatory intermediaries [73,74]. Antioxidant and radical scavenging activities, control of cellular activities of inflammation-related cells, modulation of the activities of arachidonic acid metabolism enzymatic phospholipase A2 (PLA2), COX-2, iNOS, and lipoxygenase (LOX), activation of the production of other proinflammatory molecules, and modulation of proinflammatory gene expression have all been described as mechanisms that explain the anti-inflammatory activity of flavonoids [75]. Lemon juice’s antioxidant properties and its ability to prevent TNF-α production and interferon may explain the lower levels of inflammatory factors in rats treated with the LPE extract [76].

Furthermore, the anti-inflammatory influence of RES on the proinflammatory mediators TNF-α, NF-kB, as well, as COX-2 has been linked to its mode of action, which involves numerous targets, including signal transducer and activator of transcription 3 (STAT3), MAPK14, AKT, mitogen-activated protein kinase 1/3 (MAPK1/3; ERK1/2), protein kinase C, MAPK14 (p38), ribosomal protein S6 kinase beta 2, and peroxisome proliferator-activated receptors (PPAR) gamma [77,78]. A parallel study showed that the anti-inflammatory actions of RES have been shown to be mediated through various molecular targets, one being the sirtuin-1 (SIRT). SIRT-1 regulates cell growth, metabolism, physiological stress, and cell survival as an NAD+-dependent family III histone deacetylase [15,79]. Activation of SIRT1 led to decreases in proinflammatory cytokines by inhibiting NF-kB activity [80]. Consistent with these results, our findings indicated that RES lowers the proinflammatory levels of the ovary caused by CPA, and especially when combined with LPE, it strongly suppresses the highly expressed amount of TNF-α and NF-kB [35,81].

Moreover, Subbaramaiah et al. [82] and Ibrahim et al. [35] reported that RES restricts COX-2 expressions by interfering only with the pathway of arachidonic acid and reducing both cyclooxygenase production in differentiated mammary cells and the protein kinase C pathway. These findings could be linked to resveratrol’s antiproliferative and anti-apoptotic properties [83]. Also, RES has been shown to reduce inflammation and inhibit COX and thyroperoxidase activities. Curiously, Jang et al. [32] and Meng et al. [84] confirmed that polyphenol inhibited prostaglandins (PGs) production by selectively suppressing COX activity in COX-1 and thus isoenzyme’s thyroperoxidase activity. RES has been shown to diminish the gene expression activated by NF-kB, for instance, TNF-IL1, IL-6, MMPs, and Cox-2 [85].

In the current investigations, histopathological and immunohistochemical examination of the ovarian tissue of CPA-treated groups revealed interstitial cell hyperplasia of variably sized clusters of pale-staining cells that were interspersed among several follicles in numerous phases of development, including large atretic follicles, mature corpora lutea, and congested blood vessels. In addition, large polyhedral cells with pale eosinophilic granular cytoplasm and central nuclei were observed in the hyperplastic interstitial cells as well as increased cytoplasmic reactivity for TNF-α in Graafian follicles and luteum. When ovarian functions failed, these changes were seen, such as elevated sex steroid hormone levels and ovarian overexpression of TNF-α, COX-2, and iNOS, which was elucidated by the physiological findings. Our results align with related research that showed that CPA can impair the ovary by producing atrophy of primordial follicles, fast stimulation of primordial follicles, follicular atresia, vascular injury, and inflammatory processes [23,86,87].

Moreover, Çil & Mete [88] indicated that the CPA group had significantly fewer follicles than the other groups. The substantial reduction in developing primordial follicles in CPA-stimulated ovarian tissue could be attributed to many factors. CPA may contribute to follicle loss by promoting cell death pathways [88], which may be one of the key mechanisms that causes the depletion of the primordial reserve [88]. 

Our exploration proved that treatment with LPE and RES reduced the toxic effects of CPA on ovarian tissue performance, primarily by minimizing follicular cell apoptosis, restoring ovarian stroma, reducing inflammatory edema, and enhancing ovarian reserve maintenance. Our deductions coincide with previous studies with similar findings where histopathological changes in ovarian tissue induced by CPA were nearly reversed, resulting in a nearly normal ovarian architecture [35,54,89]. Our findings also showed that the combination of LPE and RES represents a novel therapeutic strategy for preventing CPA-induced POF.

## 4. Materials and Methods

### 4.1. Drugs and Chemicals

Cyclophosphamide & resveratrol were provided by Sigma Chemical Company (St. Louis, MO, USA 4678992, 554325, respectively). All additional reagents, diluters, and chemicals utilized in the analysis met international quality requirements.

### 4.2. Lemon Peel Extract Preparation

Lemon peels were peeled with a grater and dried for two days at room temperature in the air [90]. Two g of dry peels were combined with 100 mL distilled H_2_O for 1 h at 95 °C. Large particles were removed by cooling the extract to 25 °C and filtering it with filter paper. The extract had a pale golden color. For future investigations, the extract was kept in the refrigerator at −4 °C. HPLC, and gas chromatography–mass spectrometry (GC–MS) analysis was estimated in lemon peel extract (see Supplementary File).

### 4.3. Experimental Animals

Housing and care of animals were conducted at the National Research Centre, Giza, Egypt. The rats were housed in polypropylene cages (seven per cage) under pathogen-free settings that included accurate lighting (12 h light/12 h dark cycle), virtual humidity (30–50%), and temperature (18–22 °C). They were nourished by typical research laboratory rat feed and had unlimited access to water. The animals were given a week to adjust to their new environments before the experiment began. All experimental protocols followed the criteria for animal experimentation, which was authorized by the Ethical Committee of Faculty of pharmacy, Suez Canal University (registration number 202203RA2). 

#### 4.3.1. Induction of POF

POF was produced by injecting CPA at a dose of 200 mg/kg intraperitoneally on day 1 and then 8 mg/kg once daily for 14 consecutive days in female rats [91].

#### 4.3.2. Study Design

A total of 35 female Wister albino rats ranging from 200–240 g, aged from 3–4 months, were split into five treatment groups (seven rats/group) corresponding to the current experimental communications link: Group I, negative control group (control): the rats were given the solvent (salt-water) daily. In Group II, the positive control group (CPA), the rats were injected with cyclophosphamide IP for 14 days. Group III (CPA + LEP): the rats received cyclophosphamide IP for the following 14 days and then were administered lemon peel extract at a dosage of 50 mg/kg b.wt./day time for a period of 3 weeks orally [92]. Group IV (CPA + RES): the rats received cyclophosphamide IP for the following 14 days and then were administered resveratrol at a dosage of 10 mg/kg b.wt./day orally for 3 weeks [34]. Group V (CPA + LPE + RES): the rats received cyclophosphamide IP for the following 14 days and were treated with both LPE and RES as described above.

#### 4.3.3. Collection of Samples

At the end of the experimentation, microcapillaries were used to obtain blood samples from the retro-orbital plexus, which were then allowed to clot; then the rats were sacrificed under ketamine anesthesia. Before analysis, serum was centrifuged for 10 min at 5000× *g* rpm for 15 min and then kept at −80 °C. Subsequently, the rats were then dissected via cervical dislocation, and their ovaries were cleansed in saline. The right ovaries were stored at −80 °C for biochemical investigation, whereas the left ovaries were kept in 10% buffered formalin as a fixative for histological evaluation.

### 4.4. Biochemical Analysis

#### 4.4.1. Serum Fertility Hormones Measurements

Blood serum hormonal testing—Luteinizing Hormone (LH), Follicle Stimulating Hormone (FSH), and progesterone—was performed according to Siiteri [93]. The concentration of serum estradiol was evaluated using an enzyme-linked immunosorbent assay corresponding to the Siiteri [93] method. All hormone concentrations were quantified by the ELISA method using a kit bought from MyBioSource Income (San Diego, CA, USA), following the manufacturer’s instructions.

#### 4.4.2. Determination of Serum Oxidative Stress and Antioxidant Markers

Lipid peroxidation was evaluated by determining the MDA level by the colorimetrical method by applying commercial kits obtained from Biodiagnostic Co., Egypt (MD 2529) according to the procedure of [94]. Reduced glutathione (GSH) level and superoxide dismutase (SOD) activity were calculated using commercial kits obtained from Biodiagnostic Co., Egypt (GP 2524, SD 2521, respectively).

#### 4.4.3. Measurements of Ovarian Inflammatory Markers

The tumor necrosis factor-alpha (TNF-α) level is determined using an ELISA kit acquired from Uscn Life Science Inc. in the United States, SEA133Ra, and followed the manufacturer’s guidelines. Nuclear factor-kappa B (NF-κB) has been measured using an ELISA method with only a rat nuclear factor-kappa B ELISA kit acquired by Glory Science Company, Ltd. in the United States, following the manufacturer’s instructions, MBS453975. Cyclooxygenase-2 (COX-2) was measured by ELISA kits from MyBioSource Inc. (San Diego, CA, USA), following the manufacturer’s guidelines.

### 4.5. Histopathological Evaluation 

After the sacrifice of all rats, ovarian tissues were stored in 10% neutral buffered formalin, embedded in paraffin, rehydrated, and cut into 5 µm-thick sections using only a rotary microtome (Bioevopeak company, Shandong, China). The sections were then stained with hematoxylin and eosin (H&E) [95]and placed in Canada balsam before being examined under an Olympus light microscope (Shenzhen China) for any histological alterations.

### 4.6. Immunohistochemical Evaluation

Polyclonal antibody tumor necrosis factor-alpha (TNF-α) was immunohistochemically stained for 15 min on formalin-fixed, paraffin-embedded sections (4 µm thick) [96].

### 4.7. qRTPCR for Determination of iNOS and Casp3 Expression Levels

The change in the transcript level of induced iNOS and Casp3 genes was quantified using qRTPCR [97,98]. Total RNA was extracted from ovarian tissues using Direct-zol RNA Miniprep Plus (Cat# R2072, ZYMO Research Corp. Tucson, Arizona USA). The concentration and purity of extracted RNA were determined by spectrophotometry. The cDNA was synthesized using an AdvanCed IV for each RT-PCR kit (Cat# 12594100, Thermal Scientific Fisher, Waltham, MA, USA) and was used for reverse transcription of isolated RNA passby PCR in a single step (SYBR Green dye). The prepared response was employed in RT-PCR (Biosystem, Foster City, CA, USA). Ten µL template RNA was added to 0.5 µL SuperScript™ IV RT Mix, 25 µL 2X Platinum™ SuperFi™ RT-PCR Master Mix, 2.5 µL of each forward and reverse primer and 9.5 µL of nuclease-free water in a final volume of 50 µL. The PCR reactions followed programs: Reverse Transcription (RT), 55 °C for 10 min one cycle, RT enzyme inactivation, 95 °C for 2 min one cycle, followed by 40 rounds of 95 °C for 10 s accompanied by 55 °C for 10 s and 72 °C for 30 s, followed by a last extension of 72 °C for 5 min for one cycle. Changes in mRNA levels were quantified using GAPDH, the housekeeping gene. Data were showed as fold change relative to a negative control [99] The PCR primers for genes and reaction condition shown in Table 1

### 4.8. Statistical Analysis

Our data were shown as the mean ± standard error of the mean. One-way ANOVA was used to evaluate the data, supplemented by the Bonferroni post hoc test. The *p*-value was used to establish the relevance of the data, whereas *p* < 0.05 is considered significant. GraphPad Prism software was used to do statistical analysis (Inc., CA, USA, 2007) [100,101,102].

## 5. Conclusions

Cyclophosphamide (CPA) is one of the chemotherapeutic drugs that causes cytotoxicity on non-target, major internal organs, as the ovary that may induce ovarian failure, by indorsing apoptosis in granulosa cells leading to follicular degeneration. Its cytotoxicity is also caused due to generation of ROS, causing lipid peroxidation, mitochondrial dysfunction, apoptosis, and inflammation. Lemon is a medicinal plant that contains citric acid, minerals, ascorbic acid, flavonoids. These compounds displayed many beneficial pharmacological actions in the body as antioxidant, anti-inflammatory, anticarcinogenic actions. RES has been reported to have anti-inflammatory, antioxidant, antiproliferative, proapoptotic, anticancer, neuroprotective, antiviral, and anti-aging acts that may improve ovarian function in a rat model of POF. In our study, we noticed that CYP administration triggered an alteration in biochemical, histological, and molecular examinations in adult female rats. Our results showed that injection with CPA caused ovarian tissue damage by increasing the oxidative stress, inflammation and apoptosis as well as affecting sex hormones that regulate ovarian follicular formation, all of which led to ovarian failure in adult female rats. On the contrary, co-treatment with LPE and RES in CPA-treated female rats showed highly protective effects on the ovarian tissues and the production of sex hormones, as struggle against free radicals, reduce inflammation, and stop cell apoptosis; moreover, the histological examinations showed a restoration in the ovarian follicular formation stages. As a result, we expect that LPE and RES administration represent great applications for chemotherapy patients and will effectively treat premature ovarian failure. Further reviews are crucial to be affective in this regard in the future.

## Figures and Tables

**Table 1:** PCR primers, genes, and reaction conditions.

Gene Name—Accession No.	Gene	5-Primer Sequence-3	Tm
Induced nitric oxide synthase (NM_000625)	iNOS	Fd GCTCTACACCTCCAATGTGACC Rv CTGCCGAGATTTGAGCCTCATG	60
Caspase-3 (NM_004346.4)	Casp3	Fd AGGACTCTAGACGGCATCCA Rv CAGTGAGACTTGGTGCAGTGA	60
Glyceraldehyde-3-phosphatedehydrogenase (NM_002046)	GAPDH	Fd TGGATTTGGACGCATTGGTC Rv TTTGCACTGGTACGTGTTGAT	56

## Data Availability

Not applicable.

## Supplementary Materials

The following supporting information can be downloaded at: https://www.mdpi.com/article/10.3390/molecules28010122/s1, Supplementary S1. The chemical composition of Lemon peel extract. (A) Flavonoid HPLC Chromatograph. (B) Phenolics HPLC Chromatograph (C) GC-TSQ mass spectrometer. The total chemical composition contained 33 identified compounds. The main components consist, á-D-Glucopyranose,4-O-á-D-galactopyranosyl 10.11%, Limonene 7.67%, Oleic Acid 7.30%, Phenol, 2-ethoxy-5-(1-propenyl)- 8.07% and n-Hexadecanoic acid 5.76%.

## Sample Availability

The samples are available from the authors.
