# Supplementary material for: Ameliorative Effect of Citrus Lemon Peel Extract and Resveratrol on Premature Ovarian Failure Rat Model: Role of iNOS/Caspase-3 Pathway
Source: Molecules. 2022 Dec 23;28(1):122. doi: 10.3390/molecules28010122 (PMC9822383; doi:10.3390/molecules28010122)
Supplement: Supplementary file 1 [file molecules-28-00122-s001.zip › molecules-2055768-supplementary.pdf]

A)

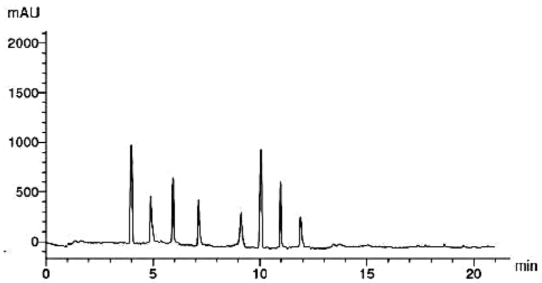

RT#	Compound	Concentration µg/ml
4.0	Naringin	10.66
5.0	Rutin	7.15
6.0	7-OH flavone	8.32
7.0	Querestin	6.78
9.0	Luteolin	4.06
10.0	Kampferol	10.58
11.0	Hisperdin	8.86
12.0	Catechin	2.56

B)

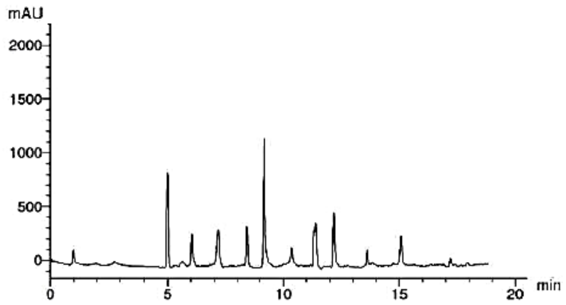

RT#	Compound	Concentration µg/ml
5.0	Syringenic	8.23
6.0	p-coumaric	2.14
7.0	Cinnamic	3.06
8.2	Caffeic	2.55
9.2	pyrogallol	13.37
10.0	Gallic	1.22
11.2	Ferulic	4.87
12.0	Salicylic	5.16
15.0	Benzoic	2.55

C)

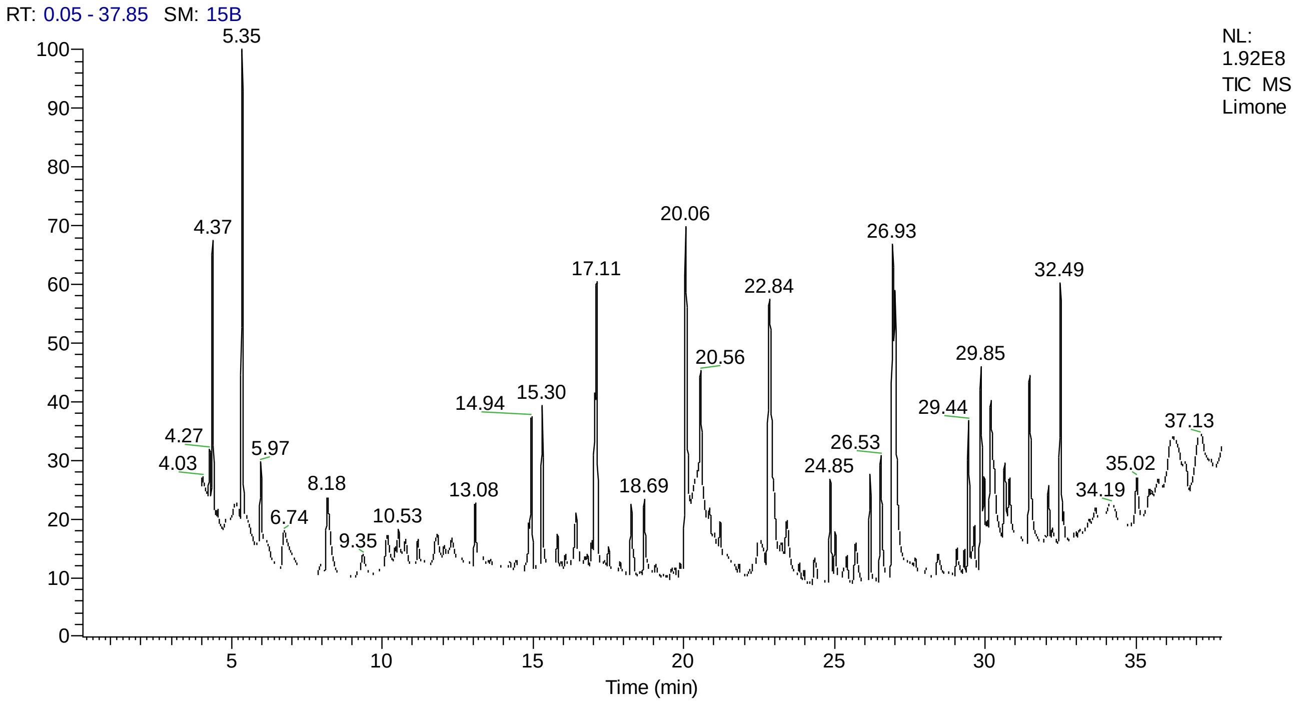

Compound number	Retention time	Compound name	Area%	Molecular weight	Molecular formula
1	4.27	(+)-SABINENE	0.78	136	$C_{10}H_{16}$
2	4.37	á-Pinene	4.18	136	$C_{10}H_{16}$
3	5.35	Limonene	7.67	136	$C_{10}H_{16}$
4	5.97	3-Carene	1.55	136	$C_{10}H_{16}$
5	8.17	4H-Pyran-4-one,	1.92	144	$C_6H_8O_4$

		2,3-dihydro-3,5-dihydroxy-6-methyl-			
6	13.07	1,2,3-PROPANETRIOL, TRIACETATE	1.73	218	C9H14O6
7	14.87	trans-Farnesol	0.59	222	C15H26O
8	14.94	Caryophyllene	2.06	204	C15H24
9	15.30	cis-à-Bergamotene	2.87	204	C15H24
10	15.81	2,6,10-DODECATRIEN-1-OL, 3,7,11-TRIMETHYL-	0.60	222	C15H26O
11	16.43	à-ylangene	1.19	204	C15H24
12	17.04	à-Farnesene	2.36	204	C15H24
13	17.11	á-Bisabolene	4.57	204	C15H24
14	18.25	ç-Elemene	1.24	204	C15H24
15	18.69	(-)-Spathulenol	1.56	220	C15H24O
16	20.06	Phenol, 2-ethoxy-5-(1-propenyl)-	8.07	178	C11H14O2
17	20.56	Tetradecane, 2,6,10-trimethyl-	2.98	240	C17H36
18	21.20	Epiglobulol	0.54	222	C15H26O
19	22.84	á-D-Glucopyranose, 4-O-á-D-galactopyranosyl-	10.11	342	C12H22O11
20	26.17	Hexadecanoic acid, methyl ester	1.90	270	C17H34O2
21	26.53	Cyperenone	2.65	218	C15H22O
22	26.93	n-Hexadecanoic acid	5.76	256	C16H32O2
23	27.00	2H-1-Benzopyran-2-one, 5,7-dimethoxy	4.65	206	C11H10O4
24	29.44	11-Octadecenoic acid, methyl ester	2.58	296	C19H36O2
25	29.85	2,5-Cyclohexadiene-1,4-dione, 2,5-bis(1,1-dimethylpropyl)-	3.73	248	C16H24O2
26	29.96	Heptadecanoic acid, 9-methyl-, methyl ester	1.07	298	C19H38O2
27	30.18	Oleic Acid	7.30	282	C18H34O2
28	30.80	Arborescin	1.40	248	C15H20O3
29	31.47	Pimpinellin	3.54	246	C13H10O5
30	32.10	9-Octadecenoic acid (Z)-, 2-hydroxy-1-(hydroxymethyl)ethyl ester	1.01	356	C21H40O4
31	32.49	4a,7,7,10a-Tetramethyldodecahydro benzo[f]chromen-3-ol	4.69	266	C17H30O2
32	35.02	2-[4-methyl-6-(2,6,6-trimethylcycloh ex-1-enyl)hexa-1,3,5-trienyl]cyclohe x-1-en-1-carboxaldehyde	1.25	324	C23H32O
33	36.22	Ethyl iso-allocholate	0.83	436	C26H44O5

Supplementary S1. The chemical composition of Lemon peel extract. (A) Flavonoid HPLC Chromatograph. (B) Phenolics HPLC Chromatograph (C) GC-TSQ mass spectrometer. The total chemical composition contained 33 identified compounds. The main components consist, á-D-Glucopyranose,4-O-á-D-galactopyranosyl 10.11 %, Limonene 7.67 %, Oleic Acid 7.30%, Phenol, 2-ethoxy-5-(1-propenyl)- 8.07% and n-Hexadecanoic acid 5.76%.
